# A combination of 5/6‐nephrectomy and unilateral ureteral obstruction model accelerates progression of remote organ fibrosis in chronic kidney disease

**DOI:** 10.1096/fba.2023-00045

**Published:** 2023-08-19

**Authors:** Kyoka Homma, Yuki Enoki, Sato Uchida, Kazuaki Taguchi, Kazuaki Matsumoto

**Affiliations:** ^1^ Division of Pharmacodynamics Keio University Faculty of Pharmacy Tokyo Japan

**Keywords:** extrarenal tissue, fibrosis, chronic kidney disease, fibrosis, indoxyl sulfate, transforming growth factor‐β, uremic toxin

## Abstract

Chronic kidney disease (CKD) involves progressive renal fibrosis, which gradually reduces kidney function and often causes various complications in extrarenal tissues. Therefore, we investigated fibrogenesis in extrarenal tissues (heart, liver, and lungs) in different experimental CKD models, such as the 5/6‐nephrectomy (5/6 Nx), unilateral ureteral obstruction (UUO), and a combination (2/3 Nx + UUO). We evaluated the degree of fibrogenesis in kidneys and extrarenal tissues by histological analysis and quantification of fibrosis‐related gene and protein expression. To elucidate the fibrosis mechanisms observed in 2/3 Nx + UUO mice, we evaluated the effect of indoxyl sulfate (IS), a typical uremic toxin accumulated in CKD, and transforming growth factor‐β (TGF‐β), a fibrosis‐related factor, on fibrosis using human hepatoma (HepG2) and RAW264.7 cells. A significant decline in renal function was observed in the 5/6 Nx and 2/3 Nx + UUO models, whereas a significant increase in renal fibrosis was observed only in the obstructed kidneys. Notable amount of fibrosis was induced in the liver and heart in the 2/3 Nx + UUO model, with the induction of macrophage infiltration and increased tissue IS and TGF‐β levels. In agreement with the results of in vivo experiments, co‐stimulation with IS, TGF‐β, and macrophage‐conditioned medium increased the expression of fibrogenic genes in HepG2 cells. We demonstrated that the 2/3 Nx + UUO model induced both loss of renal function and renal fibrosis in the earlier stages, providing a novel CKD model that induces remote organ fibrosis in a shorter time.

## Graphical abstract

We investigated fibrogenesis in extrarenal tissues (heart, liver, and lungs) in different experimental CKD models, such as the 5/6‐nephrectomy, unilateral ureteral obstruction (UUO), and a combination (2/3 Nx + UUO). Notable amount of fibrosis was induced in the liver and heart in the 2/3 Nx + UUO model, with the induction of macrophage infiltration and increased tissue IS and TGF‐β levels. The 2/3 Nx + UUO model provides a novel CKD model that induces remote organ fibrosis in a shorter time.

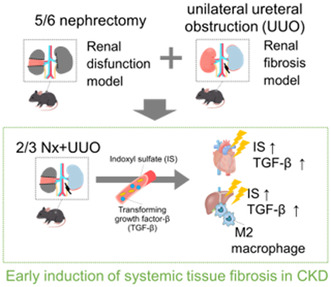

## 1 INTRODUCTION

Chronic kidney disease (CKD) is a major public health concern affecting approximately 843 million individuals worldwide.^1^, ^2^ CKD progression often leads to end‐stage renal disease (ESRD) and requires the treatment with dialysis or kidney transplantation. The kidneys play an essential role in the elimination of many metabolites; therefore, reduced kidney function leads to extrarenal complications such as respiratory failure^3^ and cardiovascular disease.^4^, ^5^ These extrarenal manifestations are the main causes of mortality. The complications also decreases the quality of life and increases mortality in patients with CKD.^6^ The proportion of deaths from cardiovascular diseases is the highest in CKD; therefore, extensive experiments are needed to prevent extrarenal injury associated with CKD pathology.

There are two possible explanations for CKD progression. First is increased renal tissue fibrosis.^7^ Second involves uremic toxin accumulation induced by decreased renal function.^8^, ^9^ Previous studies have demonstrated that fibrogenic factors and uremic toxins can get transmitted to remote organs in CKD mouse models.^10^, ^11^, ^12^, ^13^, ^14^ Extrarenal tissue fibrosis is driven by chronic inflammation through the accumulation of reactive oxygen species, other oxidative stress‐related mediators, and cytokines; thus, leading to the recruitment of multiple inflammatory cells (lymphocytes, polymorphonuclear leukocytes, and macrophages).^12^, ^15^, ^16^ Macrophages play a major role in tissue fibrosis driven by transforming growth factor‐β (TGF‐β). Liver fibrosis is associated with portal hypertension and stem cell ischemia that can lead to liver cirrhosis.^17^ Liver cirrhosis is also involved in acute kidney injuries, such as acute kidney injury hepatorenal syndrome (AKI‐HRS) or CKD‐HRS.^18^ Lung fibrosis destroys the normal alveolar architecture and disrupts gas exchange, leading to respiratory failure.^19^ Myocardial fibrosis is associated with left ventricular dysfunction and arrhythmia, ultimately leading to heart failure.^20^ Therefore, the progression of fibrosis is observed not only in the kidney but also in systemic organs, and is an important pathology of concern in CKD.

Experimental animal models of CKD are important to elucidate its pathology and contribute to the development of new therapeutic strategies. CKD is diagnosed based on the observation of kidney injury and loss of kidney function with an index glomerular filtration rate (GFR) of <60 mL/min/1.73 m^2.^ ^21^ Thus, CKD models can be generated by physical nephrectomy or nephropathic drug administration to reduce kidney function. Subtotal nephrectomy, also known as the 5/6 nephrectomy (5/6 Nx) model, is the gold standard CKD model. The 5/6 Nx model acts by physically reducing the number of nephrons and inducing a pressure load on the kidneys, which increases the filtration of blood per nephron. This model mimics progressive renal failure in humans^22^ and induces renal fibrosis, accumulation of uremic toxins, and expression of TGF‐β.^23^, ^24^ However, since the model takes several months to induce renal fibrosis, it takes more time to investigate the cross talk between the kidneys and remote organs. In previous reports, accelerated progress in experimental CKD models, such as high protein or phosphate dietary overload in the 5/6 Nx model, has been established.^25^, ^26^ These models can simulate the CKD pathology over a short period by accelerating CKD progression. However, in patients with CKD, protein and phosphate intake is limited and controlled by drug treatment. Thus, accelerated CKD pathophysiology due to protein or phosphate overload does not reflect the extrarenal manifestations associated with clinical CKD progression, although these models can be used to evaluate the impact of these factors on kidney injury. Considering these limitations, it is essential to develop an animal model that reflects the accelerated progression of extrarenal injury in clinical CKD.

The unilateral ureteral obstruction (UUO) model is a fibrosis‐specific CKD model that induces severe tubulointerstitial fibrosis. This model can demonstrate renal fibrosis in a short time course because completely obstructed kidneys develop hydronephrosis.^27^ However, this model deviates from clinical CKD pathology in that it does not reflect a decrease in renal function, accumulation of uremic toxins, or other renal excretory factors. Several studies have evaluated remote organ fibrosis using UUO models.^12^, ^16^ Both renal fibrosis and accumulation of uremic toxins due to decreased renal function occur in combination with clinical CKD pathology; therefore, the UUO model may not reflect the actual CKD pathology. Thus, the CKD‐induced extrarenal pathology could not be fully elucidated using the UUO model.

Therefore, in this study, we combined the UUO and 5/6 Nx models to demonstrate accelerated extrarenal fibrosis associated with CKD progression. Cross‐sectional assessment of the susceptibility and differences in remote organs to fibrosis in different CKD models is very informative for elucidating the effects of remote organ damage on CKD pathology. In this study, we conducted a cross‐sectional evaluation of the effects of UUO, 5/6 Nx, and a novel, complicated CKD model (2/3 Nx + UUO) on various organs. We evaluated the effects of extrarenal fibrosis, focusing on the fibrogenic factors associated with progressive kidney injury and uremic toxin accumulation due to decreased renal function, to investigate the cross talk between the kidney and remote organs.

## 2 METHODS

### 2.1 Ethical approval

All animal experiments were performed in accordance with protocols approved by the Institutional Animal Care and Use Committee of Keio University (Approval number: A2022‐60, A2022‐037, A2023‐008).

### 2.2 Cell culture

HepG2 (RRID:CVCL_0027) and RAW 264.7 cells (RRID:CVCL_4478) were purchased from the RIKEN BioResource Cell Bank (Ibaraki, Japan). Both the cell lines were cultured in Dulbecco's modified eagle medium (DMEM) supplemented with 10% fetal bovine serum (Thermo Fisher Scientific Inc.), and 100 U/mL penicillin and 100 μg/mL streptomycin (Nacalai Tesque) at 37°C in a humidified 5% CO_2_ incubator. Cells were seeded in a 96‐well plate and incubated at 37°C in a humidified 5% CO_2_ incubator. After overnight culture, the cells were grown to 70%–80% confluence and treated with 1 mM indoxyl sulfate potassium salt (Santa Cruz Biotechnology), 10 ng/mL recombinant human TGF‐β (R&D Systems, Inc.), or recombinant mouse TGF‐β (R&D Systems, Inc.). A macrophage culture stimulated by 1 mM indoxyl sulfate (IS) and 10 ng/mL TGF‐β was generated after 72 h of incubation.

### 2.3 cDNA synthesis and quantitative real‐time RT‐PCR

Total RNA was extracted from the homogenized organ samples (kidney, liver, heart, and lungs) using RNAiso PLUS (TaKaRa Bio Inc.) according to the manufacturer's instructions. The concentration and purity of the RNA extracts were determined by measuring the absorbance at 260 and 280 nm. Complementary DNA (cDNA) was synthesized using ReverTra Ace qPCR RT Master Mix (TOYOBO Inc.). The culture cells were lysed, and cDNA was synthesized using a Superprep II^Ⓡ^ Cell Lysis RT Kit for qPCR (TOYOBO Inc.) according to the manufactures' instructions. The cDNA synthesized from both the organ samples and cultured cells were subjected to quantitative PCR with a CFX96 real‐time PCR system (Bio‐Rad Laboratories, Inc.) using a KOD SYBR qPCR Mix (TOYOBO Inc.). We used the ΔΔCt method to analyze relative expression. Primers used for qPCR are listed in Table S1. The expression levels of the target genes were normalized to the expression levels of glyceraldehyde 3‐phosphate dehydrogenase (GAPDH), β‐actin, or TATA binding protein (TBP), as appropriate.

### 2.4 Animal experiments

**FIGURE 1:**
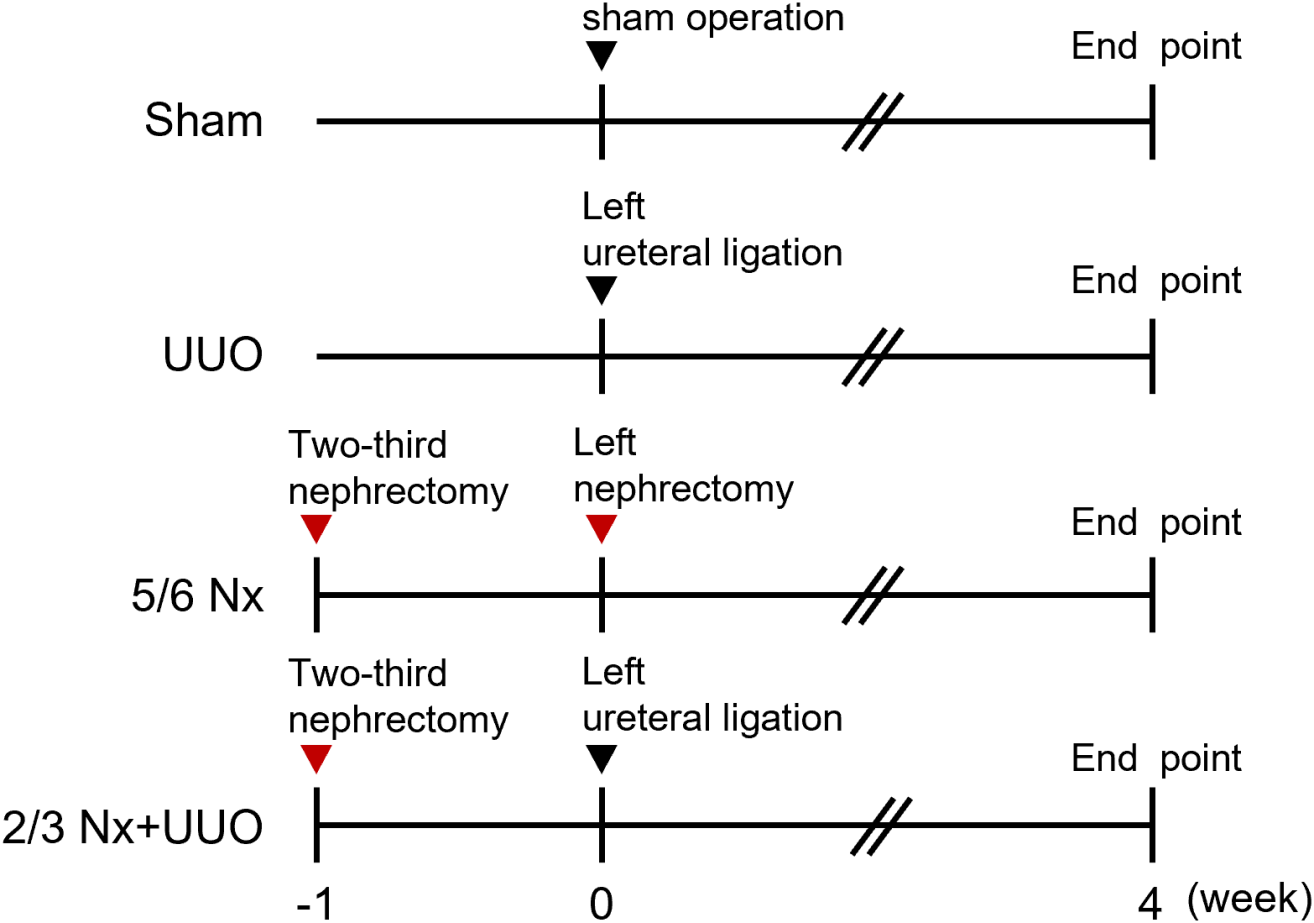
Experimental scheme. Twelve‐week‐old C57BL/6J male mice were randomly allocated to sham, unilateral ureteral obstruction (UUO), 5/6 nephrectomy (5/6 Nx), or a combination of 2/3 nephrectomy and UUO (2/3 Nx + UUO) groups.

A detailed experimental scheme is presented in Figure 1. Six‐week‐old male (C57BL/6JJmsSlc mice, *n* = 36, RRID:MGI:5488963) were purchased from Japan SLC Inc. and housed under a 12 h light/dark cycle. All animals were maintained on standard chow and water. UUO, 5/6‐nephrectomy (5/6 Nx), and combination (2/3 Nx + UUO) mouse models were generated as previously described.^28^ For the UUO surgery, the left ureter was ligated with silk at two points. In the 5/6 Nx surgery, 2/3 of the right kidney was removed, and after 1 week, the left kidney was completely removed. For 2/3 Nx + UUO surgery, 2/3 of the right kidney was removed, and after 1 week, the left ureter was ligated with silk at two points. All surgeries were performed under 3% gaseous isoflurane anesthesia. Plasma and organ samples (kidneys, liver, heart, and lungs) were collected 4 weeks after the final surgery.

### 2.5 Serum biochemistry test

Plasma levels of blood urea nitrogen (BUN), creatinine, aspartate aminotransferase (AST), and alanine aminotransferase (ALT) were measured using Fuji DRI‐CHEM 7000 and DRI‐CHEM slide systems (FUJIFILM) according to the manufacturer's instructions.

### 2.6 Western blot analysis

Total protein was extracted from all the organs samples (kidney, liver, heart, and lungs) using RIPA buffer containing 150 mM NaCl, 1% Nonidet P‐40, 10 mM Tris‐HCl (pH 7.4), and 1% protease inhibitor cocktail (Nacalai Tesque) on ice. The lysates were collected after centrifugation at 13,000 *g* at 4°C for 10 min to remove the debris. Equal amounts of protein from organ samples were used for western blot analysis. The appropriate amount of extracted protein from all the samples was heated with a sample buffer containing dithiothreitol at 100°C for 3 min and subjected to 10% sodium dodecyl sulfate‐polyacrylamide gel electrophoresis. Proteins were electro‐transferred onto polyvinylidene fluoride membranes. The membranes were blocked with 5% skim milk and incubated with primary antibodies for type 1 collagen (Southern Biotech Cat# 1310‐01, RRID:AB_2753206), α‐SMA (Abcam Cat# ab240654, RRID:AB_2922779), GAPDH (Proteintech Cat# HRP‐60004, RRID:AB_2737588), β‐actin (Cell Signaling Technology Cat# 4967, RRID:AB_330288), followed by incubation with horseradish peroxidase‐conjugated secondary antibodies at the room temperature for 1 h. The intensity of each band was detected using an Amersham Imager 600 and quantified using ImageJ software. Band intensities were normalized to those of GAPDH or β‐actin.

### 2.7 High‐performance liquid chromatography (HPLC) analysis

The levels of IS in the plasma and the organ samples were measured using HPLC, as described previously.^28^ Briefly, plasma was mixed with acetonitrile (1:9) and centrifuged at 12,000 *g* for 10 min. Organ‐lysed samples (kidneys, liver, heart, and lungs) were mixed with acetonitrile (1:3) and centrifuged at 12,000 *g* for 10 min. The supernatant was collected and mixed with ultrapure water at (1:1). The prepared samples (20 μL), from plasma and the organ samples, was loaded onto HPLC system consisting of a column (Unison UK‐C18:3 μm, 250 mm × 4.6 mm), a fluorescence detector, and a pump (SHIMADZU Co.). The mobile phases were composed of 0.2 M acetate buffer (pH 4.0)‐acetonitrile (3:1). The flow rate was set at 0.8 mL/min. The fluorescence detector excitation and emission wavelengths were set to 280 and 375 nm, respectively.

### 2.8 Enzyme‐linked immunosorbent assay (ELISA)

Total protein was extracted on ice using RIPA buffer containing 150 mM NaCl, 1% Nonidet P‐40, 10 mM Tris–HCl (pH 7.4), and 1% protease inhibitor cocktail (Nacalai Tesque). The lysates were collected after centrifugation at 13,000 *g* at 4°C for 10 min. Equal amounts of protein from organ samples (kidney, liver, heart, and lungs) and plasma were used for the analysis. The levels of TGF‐β in plasma and organ samples were measured using a Duoset ELISA kit (R&D Systems, Inc., cat#:1679‐05) in accordance with the manufacturer's instructions.

### 2.9 Histological staining

All paraffin‐embedded sections were sectioned at 4‐μm thickness. Periodic acid–Schiff (PAS) staining was performed according to standard procedures. Sirius Red staining was performed using a Picro‐Sirius Red Stain Kit (for collagen) (ScyTek Laboratories, Inc.). For immunofluorescence staining, the sections were incubated with primary antibodies against α‐SMA (Abcam; cat#: ab7817), F4/80 (ABclonal, cat#: A18637), CD206 (Proteintech, cat#:60143‐1‐Ig), and iNOS (Abcam, cat#: ab15323) for 1 h. Tissue sections were then washed and incubated with the corresponding secondary antibodies (goat anti‐mouse IgG (H + L) antibody Alexa Fluor488 cat#: A‐11001, Goat anti‐rabbit IgG (H + L) antibody Alexa Fluor594, cat#: A‐11012, and Goat anti‐rat IgG (H + L) Alexa Fluor350 cat#: A‐21093, Invitrogen). Samples were observed under a microscope (BZ‐X700 microscope; Keyence ×200 magnification). After staining, all slides were observed under a microscope (BZ‐700 microscope; Keyence), and the fibrotic area was quantified using a BZ‐X analyzer (Keyence).

### 2.10 Statistical analysis

Student's *t*‐test was used to evaluate significant differences between the two groups. Multiple surgery groups were compared using the Dunnett's test to evaluate significant changes compared with the control group. Multiple treatment groups were compared using the Tukey's multiple comparison test. A one‐way ANOVA was performed before the multiple comparison test for comparing more than two groups. Statistical significance was set at *p <* 0.05. The relationship between TGF‐β and indoxyl sulfate levels in the extrarenal tissue (liver, heart, and lungs) and serum was analyzed using Spearman's rank correlation coefficient.

## 3 RESULTS

### 3.1 Difference in kidney fibrosis intensity in the three types of CKD models

**FIGURE 2:**
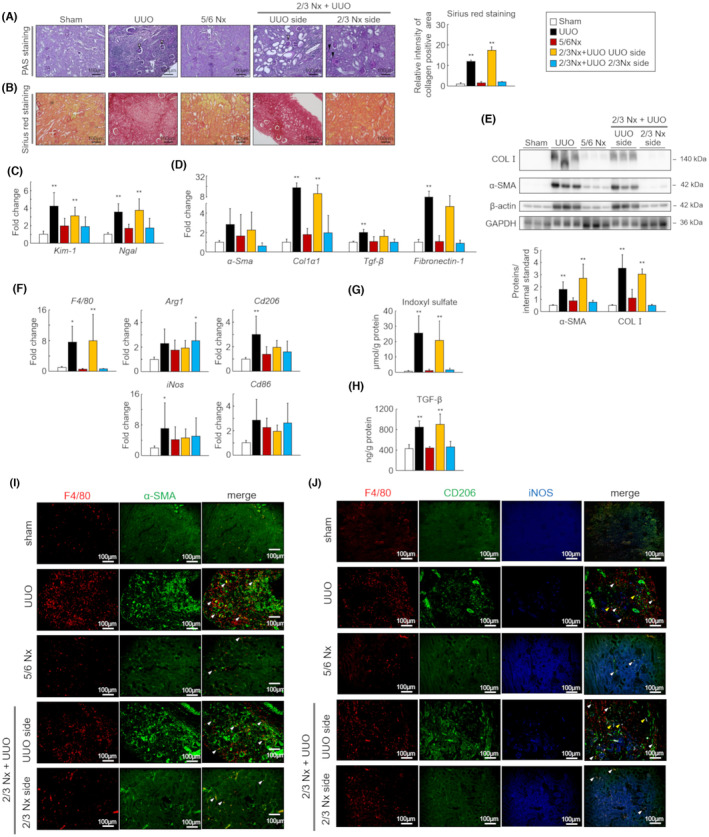
Difference in kidney fibrosis intensity in the three types of CKD models. (A) Representative images of periodic acid–Schiff‐stained kidney sections from sham, unilateral ureteral obstruction (UUO), 5/6 nephrectomy (Nx) and 2/3 Nx + UUO model mice 1 month after CKD induction. White‐out triangles indicate glomerular sclerosis. Filled triangles indicate tubular vacuolation. Scale bars indicate 100 μm. Image are expressed as 200 × magnifications. (B) Representative images of Sirius red‐stained kidney sections (top). Quantitative analysis comparing regions of renal fibrosis in the three CKD models was performed using BZ‐X analyzer (bottom). (C) mRNA expression levels of kidney injury molecule‐1 (*Kim‐1*) and neutrophil gelatinase‐associated lipocalin (*Ngal*) in the kidneys. (D) mRNA expression levels of fibrotic genes (*α‐Sma*, *Col1a1*, *Tgf‐β*, and *Fibronectin‐1*) in the kidneys. (E) Protein levels of type 1 collagen and α‐SMA as determined by western blot. (F) mRNA expression levels of macrophage‐related genes (*F4/80*, *Arg1*, *Cd206*, *iNos*, and *Cd86*) in the kidneys. (G) Concentration of indoxyl sulfate (IS) in the kidneys as measured by high‐performance liquid chromatography. (H) Transforming growth factor‐β (TGF‐β) protein levels as determined by enzyme‐linked immunosorbent assay. (I) Representative images of co‐immunostaining for macrophage (F4/80, red) and myofibroblast (α‐SMA, green) markers in kidney sections from sham, UUO, 5/6 Nx, and 2/3 Nx + UUO mouse models. (J) Representative images of the co‐immunostaining of macrophages (F4/80, red), M2 macrophages (CD206, green), and M1 macrophages (iNOS, blue) in kidney sections. White triangles indicate double‐positive M2 macrophages (F4/80+ CD206+). Scale bars indicate 100 μm. Images were captured at 200× magnification. Data are expressed as means ± SEM (*n* = 6). *p < 0.05, **p < 0.01 compared with the sham group.

First, we generated three types of CKD mouse models and investigated the differences in renal impairment 1 month after CKD induction (Figure 1). The serum levels of BUN and IS, a uremic toxin, were significantly increased in the 5/6 Nx and 2/3 Nx + UUO models compared to those in the sham group (Table 1, BUN vs sham *p =* 0.00335 and 0.00314, respectively). The serum level of TGF‐β, a master regulator of fibrosis, was significantly increased in the 2/3 Nx + UUO model compared to that in the sham group (Table 1; IS vs sham, *p =* 0.0183 and 0.0181, respectively). Next, we evaluated the intensity of the histological renal damage. Tubular cell damage was induced in the kidneys of all the three CKD models. In particular, the kidneys of 2/3 Nx + UUO mice had more strongly induced glomerular sclerosis and tubular vacuolation than the kidneys of UUO and 5/6 Nx mice (Figure 2A). In addition, the mRNA expression levels of *Kim‐1* and *Ngal*, which were acute kidney injury markers, were significantly higher in the obstructed kidney than in the sham group (Figure 2C).

**TABLE 1:** Characteristics of different types of chronic kidney disease mice.

	Sham	UUO	5/6 Nx	2/3 Nx + UUO
Body weight (g)	27.3 ± 1.8	26.3 ± 0.60	26.1 ± 0.85	25.3 ± 0.75*
BUN (mg/dL)	30.3 ± 4.7	36.9 ± 3.7	43.8 ± 7.7**	43.9 ± 5.7**
SCr (mg/dL)	0.32 ± 0.10	0.47 ± 0.20	0.42 ± 0.13	0.42 ± 0.098
AST (U/L)	39.3 ± 6.9	45.6 ± 10	47.1 ± 9.3	47.6 ± 10
ALT (U/L)	24.0 ± 2.3	24.1 ± 2.4	21.3 ± 4.0	21.0 ± 3.5
IS (μM)	8.17 ± 4.3	12.0 ± 5.3	20.1 ± 6.6**	26.4 ± 5.9**
TGF‐β (ng/mL)	3.72 ± 1.1	4.36 ± 0.69	3.92 ± 1.5	4.4 ± 1.7*

*Note*: Data are expressed as means ± SEM (*n* = 6). *,**p < 0.05 or 0.01 compared with sham group.

Abbreviations: 5/6 Nx, 5/6 nephrectomized model; ALT, alanine aminotransferase; AST, aspartate aminotransferase; BUN, blood urea nitrogen; IS, indoxyl sulfate; SCr, serum creatinine; TGF‐β, transforming growth factor‐β; UUO, unilateral ureteral obstruction model.

To investigate the differences in the intensity of kidney fibrosis among the three CKD models, we evaluated the mRNA and protein expression levels of fibrosis‐related factors. Figure 2D shows a significantly increased the mRNA expression of *Col1α1* in the obstructed kidneys. The protein expression levels of type 1 collagen and α‐SMA were also significantly increased in obstructed kidneys (Figure 2E). We also evaluated the expression of macrophage‐related genes associated with renal dysfunction and fibrosis.^29^ In the obstructed kidney, the expression of mRNAs related to the macrophage phenotypes M1 (*iNos* and *Cd86*) and M2 (*Arg1* and *Cd206*) increased. In particular, the mRNA expression levels of *F4/80*, *iNos*, and *Cd206* were significantly increased in the UUO model (Figure 2F). The obstructed kidneys in the 2/3 Nx + UUO model also showed increased mRNA expression levels of *F4/80* and *Arg1*. Immunofluorescence staining of kidney section showed that macrophage (F4/80) and α‐SMA‐positive area was increased in UUO and UUO‐performed kidney of 2/3 Nx + UUO model (Figure 2I). In addition, we evaluated macrophage polarization by co‐immunostaining for macrophages (F4/80), M1 macrophages (iNOS), and M2 macrophages (CD206) in kidney sections. Co‐stained images showed that iNOS+ macrophages (M1) and CD206+ macrophages (M2) also increased in the UUO and UUO‐treated kidneys of the 2/3 Nx + UUO model (Figure 2J, white triangle). The tissue level of IS and TGF‐β was significantly increased in obstructed kidney compared with the sham group (Figure 2G,H).

### 3.2 Difference in liver fibrosis intensity in the three types of CKD models

**FIGURE 3:**
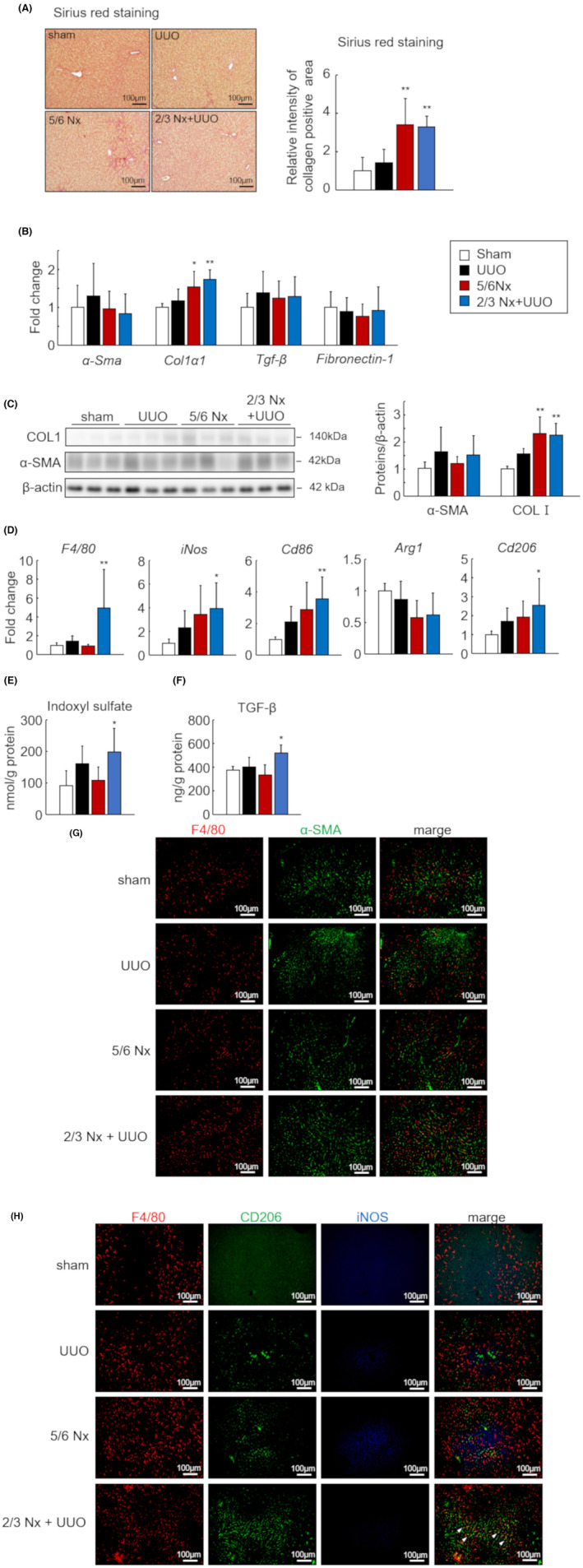
Difference in liver fibrosis intensity in the three types of CKD models. (A) Representative images of Sirius red‐stained liver sections from sham, unilateral ureteral obstruction (UUO), 5/6 nephrectomy (Nx) and 2/3 Nx + UUO model mice 1 month after CKD induction (left). Quantitative analysis comparing regions of liver fibrosis in the three CKD models was performed using BZ‐X analyzer (right). Scale bars indicate 100 μm. Images are expressed as 200× magnifications. (B) mRNA expression levels of fibrotic genes (*α‐Sma*, *Col1a1*, *Tgf‐β*, and *Fibronectin‐1*) in the livers. (C) Protein levels of type 1 collagen and α‐SMA as determined by western blot. (D) mRNA expression levels of macrophage‐related genes (*F4/80*, *Arg1*, *Cd206*, *iNos*, and *Cd86*) in the livers. (E) Concentration of indoxyl sulfate (IS) in the livers as measured by high‐performance liquid chromatography. (F) Transforming growth factor‐β (TGF‐β) protein levels as determined by enzyme‐linked immunosorbent assay. (G) Representative images of co‐immunostaining for macrophage (F4/80, red) and myofibroblast (α‐SMA, green) markers in liver sections from sham, UUO, 5/6 Nx, and 2/3 Nx + UUO mice. (H) Representative images of the co‐immunostaining of macrophages (F4/80, red), M2 macrophages (CD206, green), and M1 macrophages (iNOS, blue) in liver sections. White triangles indicate double‐positive M2 macrophages (F4/80+ CD206+). Scale bars indicate 100 μm. Images were captured at 200× magnification. Data are expressed as means ± SEM (*n* = 6). *p < 0.05, **p < 0.01 compared with the sham group.

To investigate the differences in liver fibrosis intensity among the three CKD models, we quantified the collagen accumulation area in liver sections using Sirius red staining. Collagen accumulation was significantly increased in the livers of 5/6 Nx and 2/3 Nx + UUO mice compared with that in the sham group (Figure 3A). Based on the histological findings, the mRNA and protein expression of type 1 collagen significantly increased in the 5/6 Nx and 2/3 Nx + UUO models (Figure 3B,C). Furthermore, we evaluated the expression levels of macrophage‐related genes in the liver tissue. The mRNA expression levels of *F4/80*, *iNos*, *Cd86*, and *Cd206* were significantly increased in the 2/3 Nx + UUO model (Figure 3D). In the liver sections, F4/80 and α‐SMA‐positive area was slightly increased in UUO and 2/3 Nx + UUO model, and α‐SMA‐positive area was located in near the F4/80 (Figure 3G). Images of macrophages co‐stained with iNOS and CD206 showed that iNOS+ macrophages (M1) were quite low and CD206+ macrophages (M2) were increased in the UUO, 5/6 Nx, and especially 2/3 Nx + UUO models (Figure 3H, white triangle). IS and TGF‐β tissue levels were significantly increased in the 2/3 Nx + UUO model compared to those in the sham group (Figure 3E,F).

### 3.3 Difference in lung fibrosis intensity in the three types of CKD models

**FIGURE 4:**
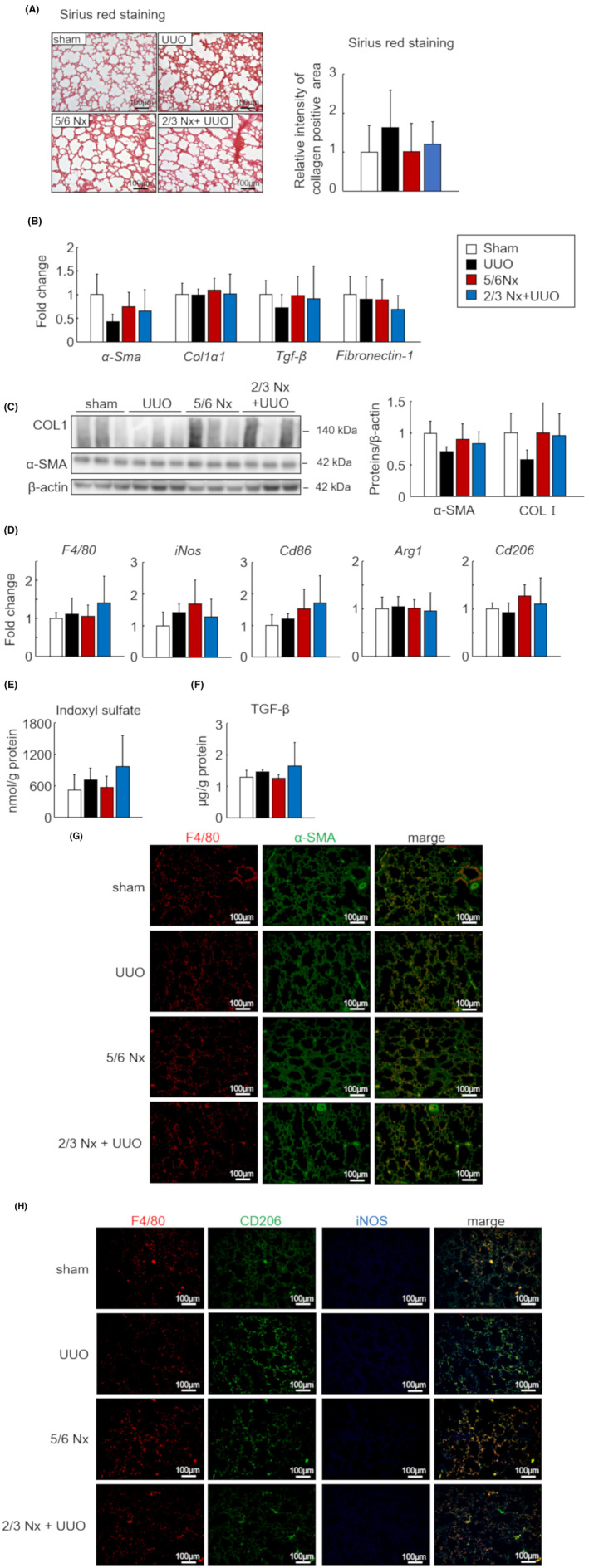
Difference in lung fibrosis intensity in the three types of CKD models. (A) Representative images of Sirius red‐stained lung sections from sham, unilateral ureteral obstruction (UUO), 5/6 nephrectomy (Nx) and 2/3 Nx + UUO model mice 1 month after CKD induction (left). Quantitative analysis comparing regions of lung fibrosis in the three CKD models was performed using BZ‐X analyzer (right). Scale bars indicate 100 μm. Image are expressed as 200× magnifications. (B) mRNA expression levels of fibrotic genes (*α‐Sma*, *Col1a1*, *Tgf‐β*, and *Fibronectin‐1*) in the lungs. (C) Protein levels of type 1 collagen and α‐SMA as determined by western blot. (D) mRNA expression levels of macrophage‐related genes (*F4/80*, *Arg1*, *Cd206*, *iNos*, and *Cd86*) in the lungs. (E) Concentration of indoxyl sulfate (IS) in the lungs was measured by high‐performance liquid chromatography. (F) Transforming growth factor‐β (TGF‐β) protein levels as determined by enzyme‐linked immunosorbent assay. (G) Representative images of co‐immunostaining for macrophage (F4/80, red) and myofibroblast (α‐SMA, green) markers in lung sections from the sham, UUO, 5/6 Nx, and 2/3 Nx + UUO mouse models. (H) Representative images of the co‐immunostaining of macrophages (F4/80, red), M2 macrophages (CD206, green), and M1 macrophages (iNOS, blue) in lung sections. White triangles indicate double‐positive M2 macrophages (F4/80+ CD206+). Scale bars indicate 100 μm. Images were captured at 200× magnification. Data are expressed as means ± SEM (*n* = 4–6). *p < 0.05, **p < 0.01 compared with the sham group.

To investigate the differences in lung fibrosis intensity among the three CKD models, we quantified the collagen area in lung sections using Sirius red staining. Lung sections in the CKD model showed a tendency for alveolar thickening; however, the collagen area did not significantly increase in any CKD model (Figure 4A). In addition, the mRNA and protein expression levels of fibrosis‐related factors did not significantly increase (Figure 4B,C). We further evaluated the expression levels of macrophage‐related genes in lung tissue; however, no significant changes were observed in either the M1 or M2 phenotype (Figure 4D). In the lung sections, no significant differences were observed in α‐SMA‐positive area and macrophage infiltration and polarization between each model (Figure 4G,H). Also, the IS and TGF‐β tissue levels were not significantly increased (Figure 4E,F).

### 3.4 Difference in cardiac fibrosis intensity in the three types of CKD models

**FIGURE 5:**
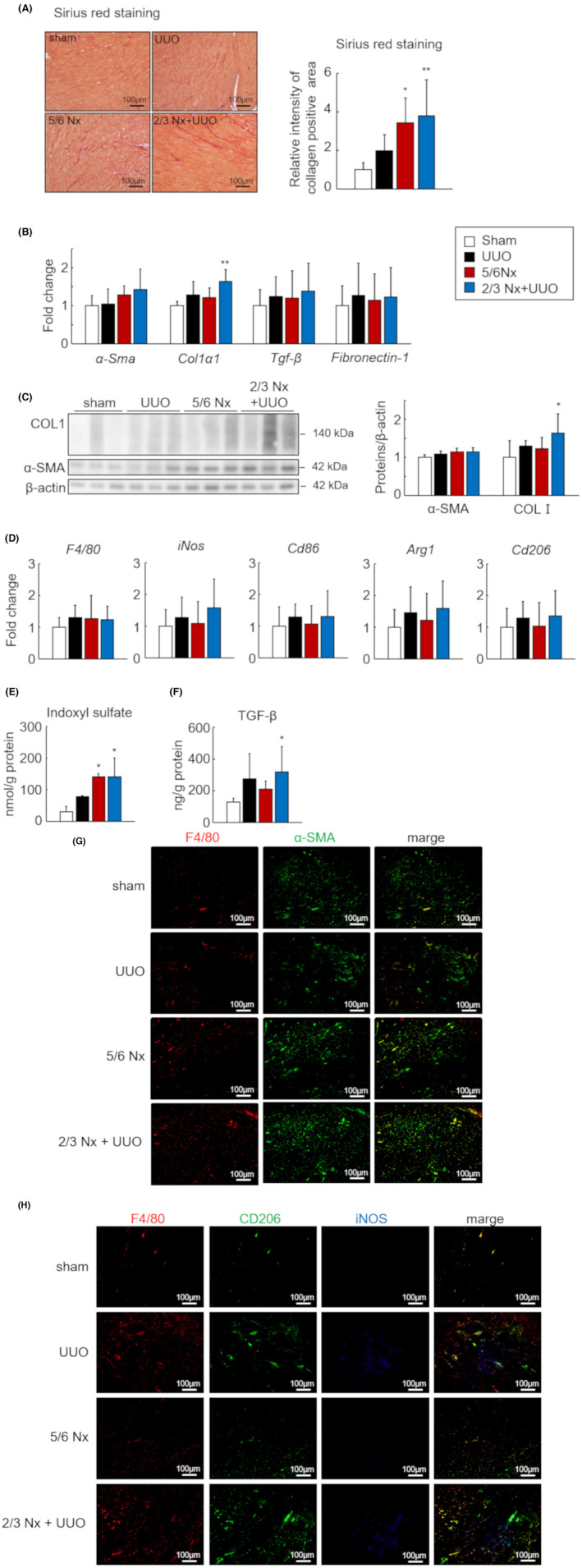
Difference in cardiac fibrosis intensity in the three types of CKD models. (A) Representative images of Sirius red‐stained myocardium sections from sham, unilateral ureteral obstruction (UUO), 5/6 nephrectomy (Nx) and 2/3 Nx + UUO model mice 1 month after CKD induction (left). Quantitative analysis comparing regions of cardiac fibrosis in the three CKD models was performed using BZ‐X analyzer (right). Scale bars indicate 100 μm. Image are expressed as 200× magnifications. (B) mRNA expression levels of fibrotic genes (*α‐Sma*, *Col1a1*, *Tgf‐β*, and *Fibronectin‐1*) in the hearts. (C) Protein levels of type 1 collagen and α‐SMA as determined by western blot. (D) mRNA expression levels of macrophage‐related genes (*F4/80*, *Arg1*, *Cd206*, *iNos*, and *Cd86*) in the hearts. (E) Concentration of indoxyl sulfate (IS) in heart as measured by high‐performance liquid chromatography. (F) Transforming growth factor‐β (TGF‐β) protein levels as determined by enzyme‐linked immunosorbent assay. (G) Representative images of co‐immunostaining for macrophage (F4/80, red) and myofibroblast (‐SMA, green) markers in myocardial sections from sham, UUO, 5/6 Nx, and 2/3 Nx + UUO model mice. (H) Representative images of co‐immunostaining of macrophages (F4/80, red), M2 macrophages (CD206, green), and M1 macrophages (iNOS, blue) in the myocardium sections. White triangles indicate double‐positive M2 macrophages (F4/80+ CD206+). Scale bars indicate 100 μm. Images were captured at 200× magnification. Data are expressed as means ± SEM (*n* = 6). *p < 0.05, **p < 0.01 compared with the sham group.

Cardiac fibrosis is one of the most severe conditions in patients with CKD. Therefore, we investigated the differences in the intensity of cardiac fibrosis among the three CKD models. The collagen area in the myocardial sections evaluated by Sirius red staining showed significant accumulation of collagen in 5/6 Nx and 2/3 Nx + UUO hearts (Figure 5A). The mRNA and protein expression levels of type 1 collagen were significantly increased in 2/3 Nx + UUO mice (Figure 5B,C). Moreover, we evaluated the expression levels of macrophage‐related genes in the myocardium; however, no significant changes were observed in any CKD model (Figure 5D). In the heart sections, α‐SMA‐positive area was increased in 5/6 Nx and 2/3 Nx + UUO model (Figure 5G). The number of CD206+ macrophages slightly increased in the heart tissue of 2/3 Nx + UUO mice (Figure 5H). IS and TGF‐β tissue levels were significantly increased in the 2/3 Nx + UUO model compared to those in the sham group (Figure 5E,F).

### 3.5 Effect of IS, TGF‐β, and macrophage on the induction of remote tissue fibrosis

**FIGURE 6:**
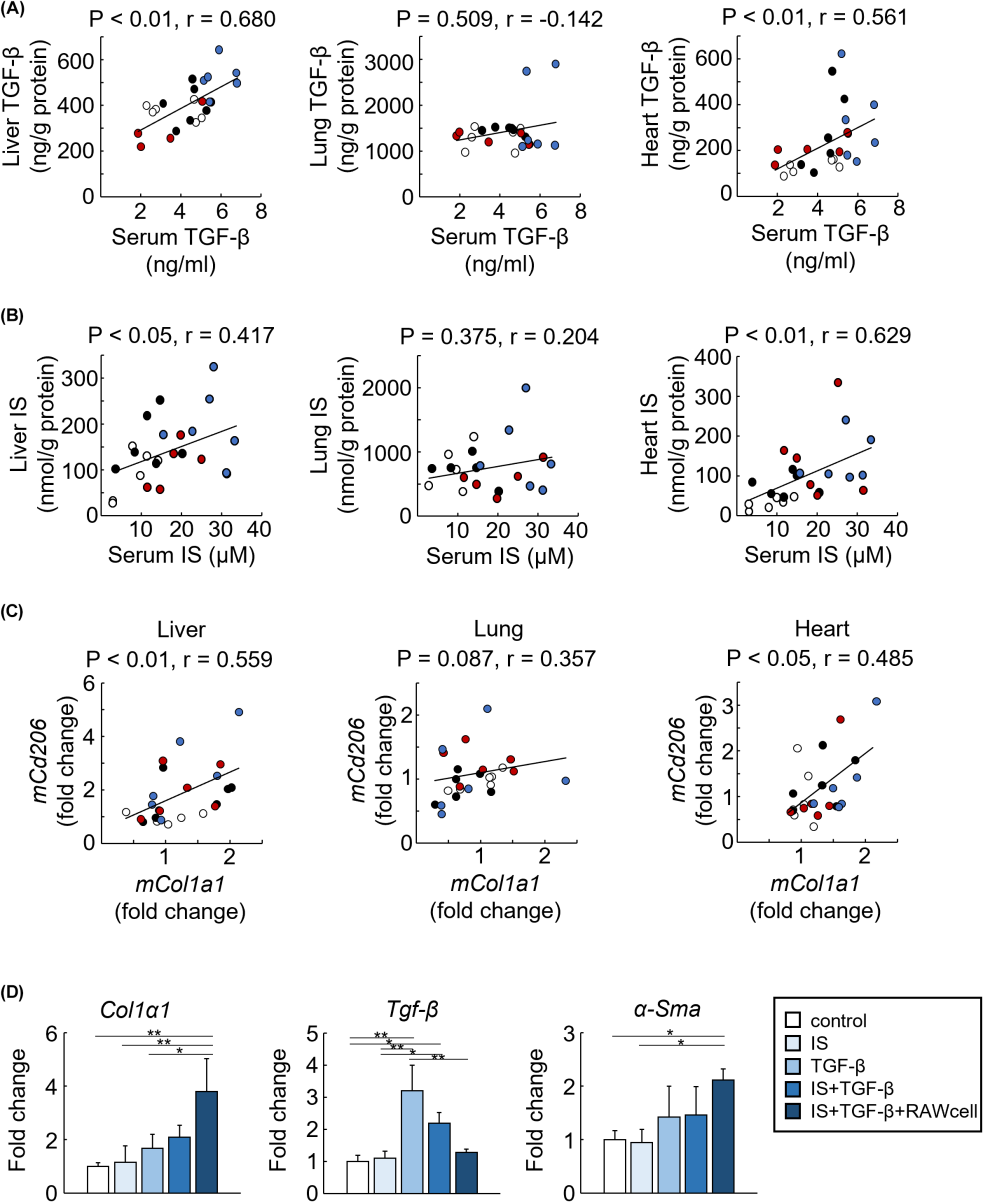
Effect of transforming growth factor‐β (TGF‐β), indoxyl sulfate (IS), and macrophage on tissue fibrosis. (A) Correlation between the TGF‐β levels in the serum and extrarenal tissue (liver, heart, and lungs). (B) Correlation between indoxyl sulfate concentration in the serum and extrarenal tissue (liver, heart, and lungs). (C) Correlation between the mRNA expression of *Col1a1* and *Cd206* in extrarenal tissue (liver, heart, and lungs). (A–C) White‐out, filled black, red, and blue circles indicate sham, unilateral ureteral obstruction (UUO), 5/6 nephrectomy (Nx), and 2/3 Nx + UUO, respectively (*n* = 4–6). (D) mRNA expression levels of fibrotic genes (*α‐Sma*, *Col1a1*, and *Tgf‐β*) in HepG2. Data are expressed as means ± SEM (*n* = 3–4). *p < 0.05, **p < 0.01.

Using cultured cells, we investigated the mechanisms underlying the significant increase in liver fibrosis observed in 2/3 Nx + UUO mice. TGF‐β and IS levels in the liver and heart were strongly correlated with serum levels (Figure 6A,B). Moreover, the mRNA levels of *Col1α1* and *Cd206* in the liver and myocardium were strongly correlated (Figure 6C). Therefore, we evaluated the effects of IS, TGF‐β, and macrophage infiltration on liver fibrosis. The mRNA expression of *Col1α1* and *α‐Sma* was substantial increased in the coculture of IS, TGF‐β, and the culture supernatant derived from macrophages stimulated with IS and TGF‐β (Figure 6D).

## 4 DISCUSSION

The pathology of CKD is characterized by loss of renal function and renal tubulointerstitial fibrosis. In this study, histopathological and biological analyses showed that the liver and heart are particularly susceptible to fibrosis in CKD. The mechanism of the early induction of fibrosis in the liver and heart prior to lung injury involves the difference between the organ‐specific accumulation of IS and TGF‐β, the key pathological factors in CKD. Moreover, this study revealed that remote organ fibrosis in 2/3 Nx + UUO model developed within a short time and was induced by the early accumulation of IS and TGF‐β and induction of macrophage infiltration.

Notably, there is pathological cross talk between the kidneys and other organs, such as the heart, liver, and lungs. Previous studies have reported that the CKD pathology induces extrarenal fibrosis associated with various factors. For heart pathophysiology in CKD, uremic cardiomyopathy induces inflammation,^16^ fibrosis,^13^, ^30^ and hypertrophy^31^ in myocardial tissue.^32^ Increases in systemic oxidative stress^14^ and pro‐inflammatory cytokines^12^ contribute to the development of lung fibrosis in patients with CKD. Liver fibrosis in CKD is induced by the onset of nonalcoholic fatty liver disease due to metabolic disturbances.^33^, ^34^, ^35^ As renal impairment triggers extrarenal tissue fibrogenesis in CKD, systemic organ fibrosis occurs simultaneously. However, as these findings have been reported independently, the cross‐sectional systemic effect of CKD on extrarenal tissue fibrosis has not yet been described. This study showed that liver and heart fibrosis was induced in the early stages of CKD. The protein expression of type 1 collagen, a typical tissue fibrosis marker, was significantly increased in the liver and heart 1 month after CKD. Previous reports indicated that myocardial fibrosis was observed shortly after CKD surgery, similar to our findings.^11^, ^36^ Our results showed that liver fibrosis was significantly and simultaneously induced at the same time. However, the protein expression of type 1 collagen did not change in the lungs within 1 month. The kidneys and lungs are closely linked in CKD pathology; however, lung fibrosis may take a long time to develop.^16^, ^37^

In this study, we investigated the development of remote organ fibrosis using classical CKD models, such as UUO, 5/6 Nx, and their combinations. The 5/6 Nx model is generated by surgical removal of renal tissue, which leads to a decrease in the absolute number of nephrons and causes loss of renal function.^22^ In contrast, the UUO model is a CKD model in which tubulointerstitial fibrosis was induced within a short time.^22^ We demonstrated that our newly developed 2/3 Nx + UUO model induced fibrosis in remote organs such as the liver and heart more strongly during the early stages of CKD. We characterized three CKD models based on the key factors associated with CKD. In the 5/6 Nx model, renal function was decreased, accompanied by the accumulation of renally excreted factors such as uremic toxins. In the UUO model, development of renal fibrosis was accompanied by the accumulation of TGF‐β in systemic circulation. In the combined 2/3 Nx + UUO model, the concentration of both IS and TGF‐β in systemic circulation significantly increased. Moreover, the extrarenal organs that developed fibrosis showed significant increases in IS and TGF‐β levels and macrophage infiltration. Therefore, we found that a combination of nephrectomy and ureteral ligation could induce systemic CKD pathology earlier than other procedures alone.

IS are typical uremic toxins derived from tryptophan. Tryptophan is decomposed into indole in the intestinal tract by intestinal bacteria and undergoes sulfate conjugation to produce IS in the liver.^38^ IS gets quickly excreted through the urine in healthy renal function; however, it accumulates in the blood^9^ and causes renal dysfunction in various organs.^39^ IS, when taken up by cells and tissues, promotes tissue fibrosis by increasing oxidative stress^40^ and inflammation.^41^ Additionally, IS polarizes macrophages into a proinflammatory phenotype (M1 macrophages),^42^ which contributes to tissue damage.^43^ IS has been reported to be an important mediator of renal‐remote tissue cross talk via systemic circulation. In a bilateral nephrectomy‐induced acute kidney injury (AKI) model, a significant increase in IS was observed in the blood, heart, liver, and lungs.^44^ Our CKD model also showed a simultaneous increase in serum and tissue IS concentrations with loss of renal function, but not as much as in AKI. In particular, IS levels in the liver and heart correlated with serum IS levels in all three CKD models. Therefore, we confirmed the existence of IS‐mediated cross talk between the kidneys and liver or heart in the 2/3 Nx + UUO model.

TGF‐β is associated with cell differentiation, extracellular matrix production, and immune regulation, resulting in wound healing. TGF‐β signaling is activated by binding to the TGF‐β type II receptor, and this binding activated by recruitment of the TGF‐β type I receptor and dimer formation. However, excessive and sustained activation of TGF‐β signaling is a key factor in inducing fibrosis in the kidneys, liver, heart, and lungs.^45^ TGF‐β is closely related to the progression of renal dysfunction,^46^, ^47^ and is overproduced in glomerular mesangial cells, tubular epithelial cells, interstitial cells, and macrophage cells. In addition, serum levels increase in patients with CKD.^46^ Therefore, TGF‐β signaling could be used to communicate with various organs via the blood circulation. In fact, we found that the expression level of TGF‐β in the liver and heart correlated with the serum level of TGF‐β in all of three CKD models in this study. These results indicated that the TGF‐β could be a cross talk molecule between the kidneys, liver, and the heart in the 2/3 Nx + UUO model.

Macrophages are innate immune cells present in every tissue and contribute to immune function, wound healing, and homeostasis. Macrophages are polarized and play a physiological role by producing various types of cytokines depending on the environment in which they receive various stimuli.^48^ M1 macrophages have pro‐inflammatory functions, whereas M2 macrophages are noninflammatory and promote tissue remodeling.^43^ Macrophages with M2 phenotype are strongly associated with tissue fibrosis.^49^ In our study, a correlation between the mRNA expression levels of fibrosis (*Col1α1*) and M2 macrophages (*Cd206*) in both the liver and heart were observed in all the CKD models. Accordingly, we hypothesized that M2 macrophage activation is involved in extrarenal fibrosis in this CKD model.

Based on the result, the mechanism of early induction of fibrosis in the 2/3 Nx + UUO model, which most efficiently induced fibrosis, seemingly involved the accumulation of uremic toxin and TGF‐β as well as increased macrophage infiltration. Using in vitro experiments, we investigated the effects of IS, TGF‐β, and macrophage infiltration on the development of fibrosis. The mRNA expression of fibrosis‐related genes was slightly increased by single or co‐stimulation with IS and TGF‐β on HepG2 cells.

Notably, stimulation with the conditioned medium derived from macrophages strongly induced fibrosis in HepG2 cells. These results indicated that IS and TGF‐β could contribute to fibrosis through an indirect mechanism via macrophages, in addition to their direct effect on hepatocytes. Co‐stimulation with IS and TGF‐β induced macrophage polarization into M1 and M2 macrophages in vitro (Figure S1). Induction of both M1 and M2 macrophage phenotypes was observed in 2/3 Nx + UUO mouse livers and RAW264.7, respectively (Figure S1). Therefore, in 2/3 Nx + UUO model, efficient and simultaneous accumulation of IS and TGF‐β in systemic circulation as well as macrophage activation leads to early induction of remote organ fibrosis.

One limitation of this study is that the effects of the renin–angiotensin–aldosterone system and endothelin, which are important factors in CKD pathology, were not evaluated in detail. The gene expression of renin, angiotensinogen, and endothelin was evaluated (Figure S2). The mRNA expression of renin decreased, while angiotensinogen expression did not change in the kidney (Figure S2A). In contrast, the expression of endothelin‐1 (ET‐1) increased in the kidney and liver, but not in the lungs and heart. ET‐1 promotes tubular vacuolation, inflammation, and extracellular matrix accumulation, and plays an important role in the progression of CKD.^50^, ^51^ Previous reports indicated that ET‐1 expression is increased in UUO and 5/6 nephrectomy kidneys.^52^ These results indicated that increase of ET‐1 expression and accumulation of uremic toxins and TGF‐β plays an important role in fibrosis and organ damage of kidney and liver, while heart damages were induced by uremic toxins and TGF‐β in 2/3 Nx + UUO model. Second, we evaluated cardiac fibrosis but not cardiac function. Therefore, we evaluated the mRNA expression of markers associated with cardiac damage such as troponin, heart‐type fatty acid‐binding protein (h‐FABP), atrial natriuretic peptide (ANP), and brain natriuretic peptide (BNP) in the heart. Troponin mRNA expression was significantly increased in the 2/3 Nx + UUO model (Figure S3). Thus, cardiac fibrosis and functional decline were induced in the 2/3 Nx + UUO model with renal impairment.

In summary, we have revealed that the liver and heart are especially susceptible to fibrosis in the early stage of CKD because the simultaneous accumulation of IS and TGF‐β and macrophage activation is important for remote organ fibrosis. Furthermore, 2/3 Nx + UUO is an advanced CKD‐induced systemic tissue fibrosis model that efficiently induces remote organ fibrosis by mimicking the CKD‐induced fibrotic environment.

## AUTHOR CONTRIBUTIONS

Conceptualization: Kyoka Homma and Yuki Enoki. Data curation: Kyoka Homma and Sato Uchida. Formal analysis: Kyoka Homma, Sato Uchida, Yuki Enoki, and Kazuaki Taguchi. Investigation: Kyoka Homma, Sato Uchida, and Yuki Enoki. Methodology: Kyoka Homma, Yuki Enoki, Kazuaki Taguchi, and Kazuaki Matsumoto. Project administration: Kyoka Homma and Yuki Enoki. Resources: Kyoka Homma and Yuki Enoki. Supervision: Yuki Enoki, Kazuaki Taguchi, and Kazuaki Matsumoto. Validation: Kyoka Homma and Yuki Enoki. Visualization: Kyoka Homma and Yuki Enoki. Writing—original draft: Kyoka Homma and Yuki Enoki. Writing—review and editing: Sato Uchida, Yuki Enoki, Kazuaki Taguchi, and Kazuaki Matsumoto.

## FUNDING INFORMATION

This study was supported by the JSPS KAKENHI (Grant Number 18 K15069).

## CONFLICT OF INTEREST STATEMENT

The authors report no competing interests.

## Supporting information


Table S1.

Figure S1.

Data S1.

## Data Availability

The dataset generated or analyzed in this study is available from the corresponding author upon reasonable request.

## References

[1] Jager KJ , Kovesdy C , Langham R , Rosenberg M , Jha V , Zoccali C . A single number for advocacy and communication‐worldwide more than 850 million individuals have kidney diseases. Nephrol Dial Transplant. 2019;34:1803‐1805. 31566230 10.1093/ndt/gfz174

[2] Mills KT , Xu Y , Zhang W , et al. A systematic analysis of worldwide population‐based data on the global burden of chronic kidney disease in 2010. Kidney Int. 2015;88:950‐957. 26221752 10.1038/ki.2015.230 PMC4653075

[3] Salerno FR , Parraga G , McIntyre CW . Why is your patient still short of breath? Understanding the complex pathophysiology of dyspnea in chronic kidney disease. Semin Dial. 2017;30:50‐57. 27680887 10.1111/sdi.12548

[4] Gansevoort RT , Correa‐Rotter R , Hemmelgarn BR , et al. Chronic kidney disease and cardiovascular risk: epidemiology, mechanisms, and prevention. Lancet. 2013;382:339‐352. 23727170 10.1016/S0140-6736(13)60595-4

[5] Kottgen A , Russell SD , Loehr LR , et al. Reduced kidney function as a risk factor for incident heart failure: the atherosclerosis risk in communities (ARIC) study. J Am Soc Nephrol. 2007;18:1307‐1315. 17344421 10.1681/ASN.2006101159

[6] Bikbov B , Purcell CA , Levey AS , et al. Global, regional, and national burden of chronic kidney disease, 1990–2017: a systematic analysis for the global burden of disease study 2017. Lancet. 2020;395:709‐733. 32061315 10.1016/S0140-6736(20)30045-3 PMC7049905

[7] Liu Y . Renal fibrosis: new insights into the pathogenesis and therapeutics. Kidney Int. 2006;69:213‐217. 16408108 10.1038/sj.ki.5000054

[8] Vanholder R , Schepers E , Pletinck A , Nagler EV , Glorieux G . The uremic toxicity of indoxyl sulfate and p‐cresyl sulfate: a systematic review. J Am Soc Nephrol. 2014;25:1897‐1907. 24812165 10.1681/ASN.2013101062 PMC4147984

[9] Wu V‐C , Young G‐H , Huang P‐H , et al. In acute kidney injury, indoxyl sulfate impairs human endothelial progenitor cells: modulation by statin. Angiogenesis. 2013;16:609‐624. 23408148 10.1007/s10456-013-9339-8

[10] Lowenstein J , Nigam SK . Uremic toxins in organ crosstalk. Front Med. 2021;8:592602. 10.3389/fmed.2021.592602 PMC8085272 33937275

[11] Ham O , Jin W , Lei L , et al. Pathological cardiac remodeling occurs early in CKD mice from unilateral urinary obstruction, and is attenuated by Enalapril. Sci Rep. 2018;8:1‐17. 30382174 10.1038/s41598-018-34216-x PMC6208335

[12] Yang F , Chang Y , Zhang C , et al. UUO induces lung fibrosis with macrophage‐myofibroblast transition in rats. Int Immunopharmacol. 2021;93:107396. 33540244 10.1016/j.intimp.2021.107396

[13] Mawhin MA , Bright RG , Fourre JD , et al. Chronic kidney disease mediates cardiac dysfunction associated with increased resident cardiac macrophages. BMC Nephrol. 2022;23:1‐15. 35090403 10.1186/s12882-021-02593-7 PMC8796634

[14] Nemmar A , Karaca T , Beegam S , Yuvaraju P , Yasin J , Ali BH . Lung oxidative stress, DNA damage, apoptosis, and fibrosis in adenine‐induced chronic kidney disease in mice. Front Physiol. 2017;8:1‐10. 29218013 10.3389/fphys.2017.00896 PMC5703828

[15] Rockey DC , Bell PD , Hill JA . Fibrosis—a common pathway to organ injury and failure. N Engl J Med. 2015;372:1138‐1149. 25785971 10.1056/NEJMra1300575

[16] Chen G , Chang Y , Xiong Y , et al. Eplerenone inhibits UUO‐induced lymphangiogenesis and cardiac fibrosis by attenuating inflammatory injury. Int Immunopharmacol. 2022;108:108759. 35428023 10.1016/j.intimp.2022.108759

[17] Parola M , Pinzani M . Liver fibrosis: pathophysiology, pathogenetic targets and clinical issues. Mol Aspects Med. 2019;65:37‐55. 30213667 10.1016/j.mam.2018.09.002

[18] Ginès P , Solà E , Angeli P , Wong F , Nadim MK , Kamath PS . Hepatorenal syndrome. Nat Rev Dis Prim. 2018;4:23. 30213943 10.1038/s41572-018-0022-7

[19] Richeldi L , Collard HR , Jones MG . Idiopathic pulmonary fibrosis. Lancet. 2017;389:1941‐1952. 28365056 10.1016/S0140-6736(17)30866-8

[20] González A , Schelbert EB , Díez J , Butler J . Myocardial interstitial fibrosis in heart failure: biological and translational perspectives. J Am Coll Cardiol. 2018;71:1696‐1706. 29650126 10.1016/j.jacc.2018.02.021

[21] Milik A , Hrynkiewicz E . On translation of LD, IL and SFC given according to IEC‐61131 for hardware synthesis of reconfigurable logic controller. IFAC Proc. 2014;19:4477‐4483.

[22] Yang HC , Zuo Y , Fogo AB . Models of chronic kidney disease. Drug Discov Today Dis Model. 2010;7:13‐19. 10.1016/j.ddmod.2010.08.002 PMC3030258 21286234

[23] Tan RZ , Zhong X , Li JC , et al. An optimized 5/6 nephrectomy mouse model based on unilateral kidney ligation and its application in renal fibrosis research. Ren Fail. 2019;41:555‐566. 31234688 10.1080/0886022X.2019.1627220 PMC6598497

[24] Miyazaki T , Aoyama I , Ise M , Seo H , Niwa T . An oral sorbent reduces overload of indoxyl sulphate and gene expression of TGF‐β1 in uraemic rat kidneys. Nephrol Dial Transplant. 2000;15:1773‐1781. 11071964 10.1093/ndt/15.11.1773

[25] Harter HR , Karl IE , Klahr S , Kipnis DM . Effects of reduced renal mass and dietary protein intake on amino acid release and glucose uptake by rat muscle in vitro. J Clin Invest. 1979;64:513‐523. 457866 10.1172/JCI109489 PMC372146

[26] Radloff J , Latic N , Pfeiffenberger U , et al. A phosphate and calcium‐enriched diet promotes progression of 5/6‐nephrectomy‐induced chronic kidney disease in C57BL/6 mice. Sci Rep. 2021;11:1‐11. 34290280 10.1038/s41598-021-94264-8 PMC8295299

[27] Chevalier RL , Forbes MS , Thornhill BA . Ureteral obstruction as a model of renal interstitial fibrosis and obstructive nephropathy. Kidney Int. 2009;75:1145‐1152. 19340094 10.1038/ki.2009.86

[28] Enoki Y , Watanabe H , Arake R , et al. Potential therapeutic interventions for chronic kidney disease‐associated sarcopenia via indoxyl sulfate‐induced mitochondrial dysfunction. J Cachexia Sarcopenia Muscle. 2017;8:735‐747. 28608457 10.1002/jcsm.12202 PMC5659061

[29] Tang PMK , Nikolic‐Paterson DJ , Lan HYRN . Macrophages: versatile players in renal inflammation and fibrosis. Nat Rev Nephrol. 2019;15:144‐158. 30692665 10.1038/s41581-019-0110-2

[30] Lekawanvijit S , Kompa AR , Manabe M , et al. Chronic kidney disease‐induced cardiac fibrosis is ameliorated by reducing circulating levels of a non‐dialysable uremic toxin, indoxyl sulfate. PloS One. 2012;7:1‐10. 10.1371/journal.pone.0041281 PMC3400638 22829936

[31] Fujii H , Nishijima F , Goto S , et al. Oral charcoal adsorbent (AST‐120) prevents progression of cardiac damage in chronic kidney disease through suppression of oxidative stress. Nephrol Dial Transplant. 2009;24:2089‐2095. 19188341 10.1093/ndt/gfp007

[32] Mall G , Huther W , Schneider J , Lundin P , Ritz E . Diffuse intermyocardiocytic fibrosis in uraemic patients G. 1990. 39‐44. 10.1093/ndt/5.1.39 2109283

[33] Behairy MA , Sherief AF , Hussein HA . Prevalence of non‐alcoholic fatty liver disease among patients with non‐diabetic chronic kidney disease detected by transient elastography. Int Urol Nephrol. 2021;53:2593‐2601. 33675475 10.1007/s11255-021-02815-9

[34] Hara M , Tanaka S , Torisu K , et al. Non‐invasive fibrosis assessments of non‐alcoholic fatty liver disease associated with low estimated glomerular filtration rate among CKD patients: the Fukuoka kidney disease registry study. Clin Exp Nephrol. 2021;25:822‐834. 33856608 10.1007/s10157-020-02018-z

[35] Selimovic A , Mededovic S , Bijedic N , Sofic A . Biochemical parameters as predictors of underlying liver disease in patients with chronic kidney disorders. Acta Inform Medica. 2021;29:260‐265. 10.5455/aim.2021.29.260-265 PMC8800580 35197660

[36] Wang B , Zhang A , Wang H , et al. miR‐26a limits muscle wasting and cardiac fibrosis through exosome‐mediated microRNA transfer in chronic kidney disease. Theranostics. 2019;9:1864‐1877. 31037144 10.7150/thno.29579 PMC6485283

[37] Liu Z , Zhang C , Hao J , et al. Eplerenone ameliorates lung fibrosis in unilateral ureteral obstruction rats by inhibiting lymphangiogenesis. Exp Ther Med. 2022;24:1‐12. 10.3892/etm.2022.11560 PMC9468786 36160894

[38] Banoglu E , Jha GG , King RS . Hepatic microsomal metabolism of indole to indoxyl, a precursor of indoxyl sulfate. Eur J Drug Metab Pharmacokinet. 2001;26:235‐240. 11808865 10.1007/BF03226377 PMC2254176

[39] Sun YB , Han P , Liu TY , Dou GF . Pharmacokinetics and tissue distribution of Uraemic Indoxyl Sulphate in rats. J Int Pharm Res. 2020;47:296‐299.

[40] Nakano T , Watanabe H , Imafuku T , et al. Indoxyl sulfate contributes to mTORC1‐induced renal fibrosis via the OAT/NADPH oxidase/ROS pathway. Toxins (Basel). 2021;13:909. 34941746 10.3390/toxins13120909 PMC8706756

[41] Milanesi S , Garibaldi S , Saio M , et al. Indoxyl sulfate induces renal fibroblast activation through a targetable heat shock protein 90‐dependent pathway. Oxid Med Cell Longev. 2019;2019:2050183. 31178953 10.1155/2019/2050183 PMC6501427

[42] Nakano T , Katsuki S , Chen M , et al. Uremic toxin Indoxyl sulfate promotes proinflammatory macrophage activation via the interplay of OATP2B1 and Dll4‐notch signaling: potential mechanism for accelerated atherogenesis in chronic kidney disease. Circulation. 2019;139:78‐96. 30586693 10.1161/CIRCULATIONAHA.118.034588 PMC6311723

[43] Lee S , Huen S , Nishio H , et al. Distinct macrophage phenotypes contribute to kidney injury and repair. J Am Soc Nephrol. 2011;22:317‐326. 21289217 10.1681/ASN.2009060615 PMC3029904

[44] Yabuuchi N , Sagata M , Saigo C , et al. Indoxyl sulfate as a mediator involved in dysregulation of pulmonary aquaporin‐5 in acute lung injury caused by acute kidney injury. Int J Mol Sci. 2017;18:1‐9. 10.3390/ijms18010011 PMC5297646 28025487

[45] Park SA , Kim MJ , Park SY , et al. EW‐7197 inhibits hepatic, renal, and pulmonary fibrosis by blocking TGF‐β/Smad and ROS signaling. Cell Mol Life Sci. 2015;72:2023‐2039. 25487606 10.1007/s00018-014-1798-6 PMC11113926

[46] Mehta T , Buzkova P , Kizer JR , et al. Higher plasma transforming growth factor (TGF)‐β is associated with kidney disease in older community dwelling adults. BMC Nephrol. 2017;18:1‐9. 28327102 10.1186/s12882-017-0509-6 PMC5359982

[47] Miyajima A , Chen J , Lawrence C , et al. Antibody to transforming growth factor‐β ameliorates tubular apoptosis in unilateral ureteral obstruction. Kidney Int. 2000;58:2301‐2313. 11115064 10.1046/j.1523-1755.2000.00414.x

[48] Zhang F , Wang H , Wang X , et al. TGF‐β induces M2‐like macrophage polarization via SNAIL mediated suppression of a pro‐inflammatory phenotype. 2016;7. 10.18632/oncotarget.10561 PMC5239552 27418133

[49] Pan B , Liu G , Jiang Z , Zheng D . Regulation of renal fibrosis by macrophage polarization. Cell Physiol Biochem. 2015;35:1062‐1069. 25662173 10.1159/000373932

[50] Shimizu T , Hata S , Kuroda T , Mihara SI , Fujimoto M . Different roles of two types of endothelin receptors in partial ablation‐induced chronic renal failure in rats. Eur J Pharmacol. 1999;381:39‐49. 10528132 10.1016/s0014-2999(99)00535-x

[51] Neder TH , Schrankl J , Fuchs MAA , Broeker KAE , Wagner C . Endothelin receptors in renal interstitial cells do not contribute to the development of fibrosis during experimental kidney disease. Pflugers Arch Eur J Physiol. 2021;473:1667‐1683. 34355294 10.1007/s00424-021-02604-4 PMC8433107

[52] Kohan DE , Barton M . Endothelin and endothelin antagonists in chronic kidney disease. Kidney Int. 2014;86:896‐904. 24805108 10.1038/ki.2014.143 PMC4216619

