# Supplementary material for: A combination of 5/6‐nephrectomy and unilateral ureteral obstruction model accelerates progression of remote organ fibrosis in chronic kidney disease
Source: FASEB Bioadv. 2023 Aug 19;5(10):377–94. doi: 10.1096/fba.2023-00045 (PMC10551277; doi:10.1096/fba.2023-00045)
Supplement: Supplementary file 1 — Table S1. [file FBA2-5-377-s003.docx]

Supplementary Table S1 Primers used in real-time RT-PCR

| Target gene | Forward | Reverse |
| --- | --- | --- |
| Mouse | | |
| *Tbp* | 5’-GGGTATCTGCTGGCGGTTT-3’ | 5’-TGAAATAGTGATGCTGGGCACT-3’ |
| *β-actin* | 5’-GCAGGAGTACGATGAGTCCG-3’ | 5’-ACGCAGCTCAGTAACAGTCC-3’ |
| *Kim-1* | 5’-TGCCCATCTTCTGCTTGTCA-3’ | 5’-CCATCCAGGAATCTCCACTCG-3’ |
| *Ngal* | 5’-GGCCAGTTCACTCTGGGAAA-3’ | 5’-TGGCGAACTGGTTGTAGTCC-3’ |
| *α-sma* | 5’-CCAGCCATCTTTCATTGGGATGG-3’ | 5’-ATAGGTGGTTTCGTGGATGCC-3’ |
| *Col1a1* | 5’-CCCTGGTCCCTCTGGAAATG-3’ | 5’-GGACCTTTGCCCCCTTCTTT-3’ |
| *Tgf-β* | 5’-CCCGAAGCGGACTACTATGC-3’ | 5’-CATAGATGGCGTTGTTGCGG-3’ |
| *Fibronectin-1* | 5’-TGCGCTCCATTCCACCTTAC-3’ | 5’-CCAGACACAACAATGCTCCC-3’ |
| *F4/80* | 5’-CAGTGATGCTCTTCCTGATGGT-3’ | 5’-ACCCCGTCTCTGTATTCAACC-3’ |
| *Arg-1* | 5’-TGCGCCACATGAAAACCATC-3’ | 5’-TTGGGAGGAGAAGGCGTTTG-3’ |
| *Cd206* | 5’-GCACTGGGTTGCATTGGTTT-3’ | 5’-TGCAGGGTTGACATGAGACC-3’ |
| *iNos* | 5’-TCACGGAGATCAATGTGGCT-3’ | 5’-GGTGATGCTCCCAGACACTG -3’ |
| *Cd86* | 5’-TCTGCCGTGCCCATTTACAA-3’ | 5’-TGTGCCCAAATAGTGCTCGT-3’ |
| *Renin* | 5’-GCACCGCTACCTTTGAACGA-3’ | CACGGGGGAGGTAAGATTGG |
| *Angiotensinogen* | GTACAGACAGCACCCTACTT | CACGTCACGGAGAAGTTGTT |
| *Endothelin1* | GAAGTTGACGCACAACCGAG | CTCTGCCCGTCTGAACAAGA |
| *Troponin* | ATTCGCAATGAGCGGGAGAA | ACCCTCCAAAGTGCATCATGT |
| *H-fabp* | ACGGGAAACTCATCCTGACTCT | ATTGACCTTGGAGCACCCTTT |
| *Anp* | CGTCTTGGCCTTTTGGCTTC | GGTGGTCTAGCAGGTTCTTGAAA |
| *Bnp* | TTGTGGCAAGTTTGTGCTCC | TGGGCTGTAACGCACTGAAG |
| Human | | |
| *Gapdh* | 5’-CAAAATCAAGTGGGGCGATGC-3’ | 5’-GGGCAGAGATGATGACCCTTT-3’ |
| *α-sma* | 5’-CCTGAAGAGCATCCCACCCT-3’ | 5’-AGTCCAGCACGATGCCAGTTG-3’ |
| *Col1a1* | 5’-GCTCGTGGAAATGATGGTGC-3’ | 5’-ACCCTGGGGACCTTCAGAG-3’ |
| *Tgf-β* | 5’-TTGAGCCGTGGAGGGGAAAT-3’ | 5’-GGCCGGTAGTGAACCCG-3’ |

*bp*: TATA-binding protein, *Kim-1*: Kidney injury molucle-1, *Ngal*: Neutrophil gelatinase-associated lipocalin, *α-sma*: α-smooth musle actin, *Col1a1*: Collagen 1a1, *Tgf-β*: Transforming growth factor*-*β, *Arg-1*: Arginase-1, *Anp:* Atrial natriuretic peptides, *Bnp:* Brain natriuretic peptide, *Gapdh*: Glyceraldehyde-3-phosphate dehydrogenase
