# Supplementary material for: A combination of 5/6‐nephrectomy and unilateral ureteral obstruction model accelerates progression of remote organ fibrosis in chronic kidney disease
Source: FASEB Bioadv. 2023 Aug 19;5(10):377–94. doi: 10.1096/fba.2023-00045 (PMC10551277; doi:10.1096/fba.2023-00045)
Supplement: Supplementary file 2 — Figure S1. [file FBA2-5-377-s001.pptx]

## Slide 1
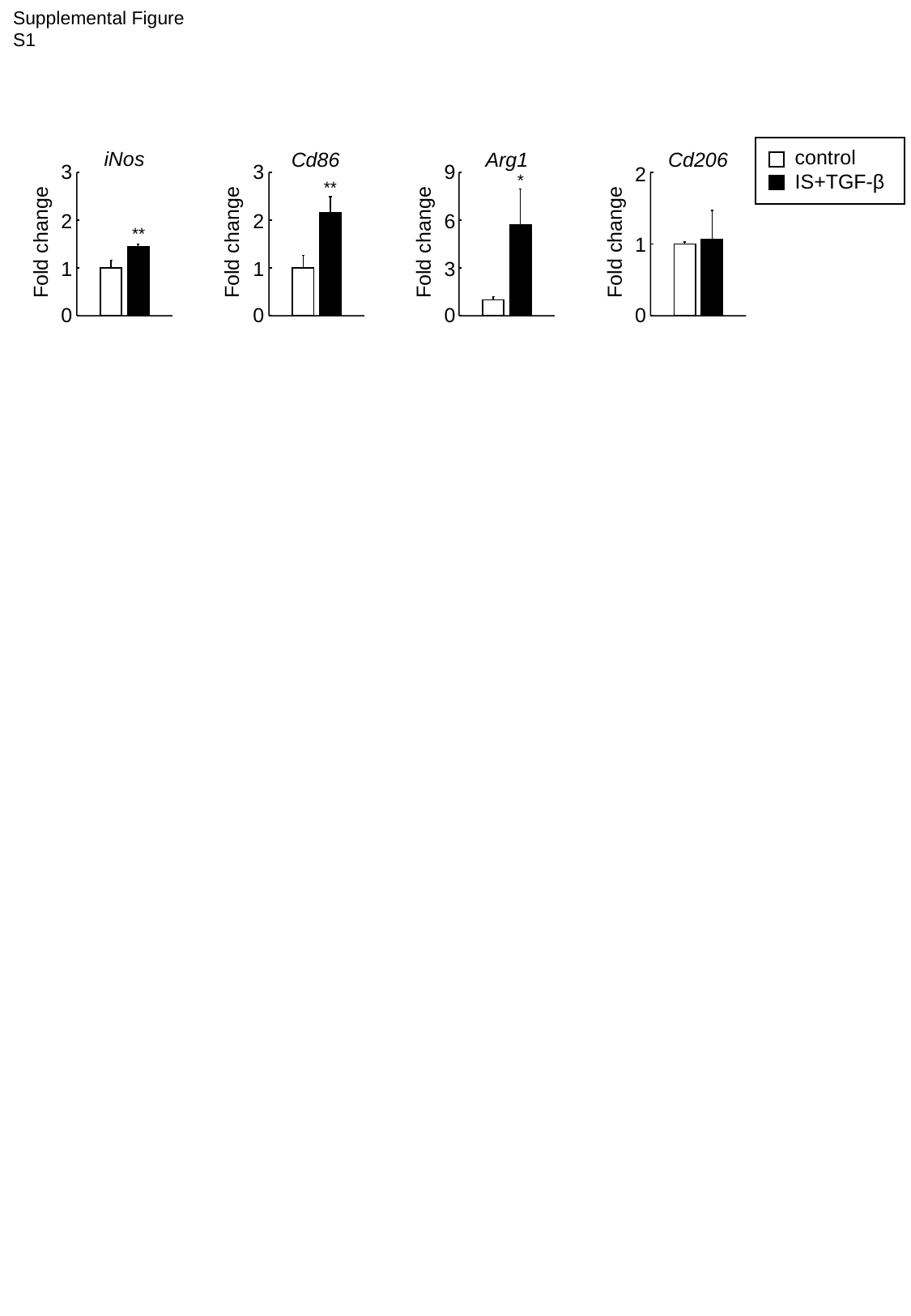

Supplemental Figure S1
control
IS+TGF-β
iNos
3
2
**
Fold change
1
0
Cd86
3
**
2
Fold change
1
0
Arg1
9
*
6
Fold change
3
0
Cd206
2
Fold change
1
0

## Slide 2
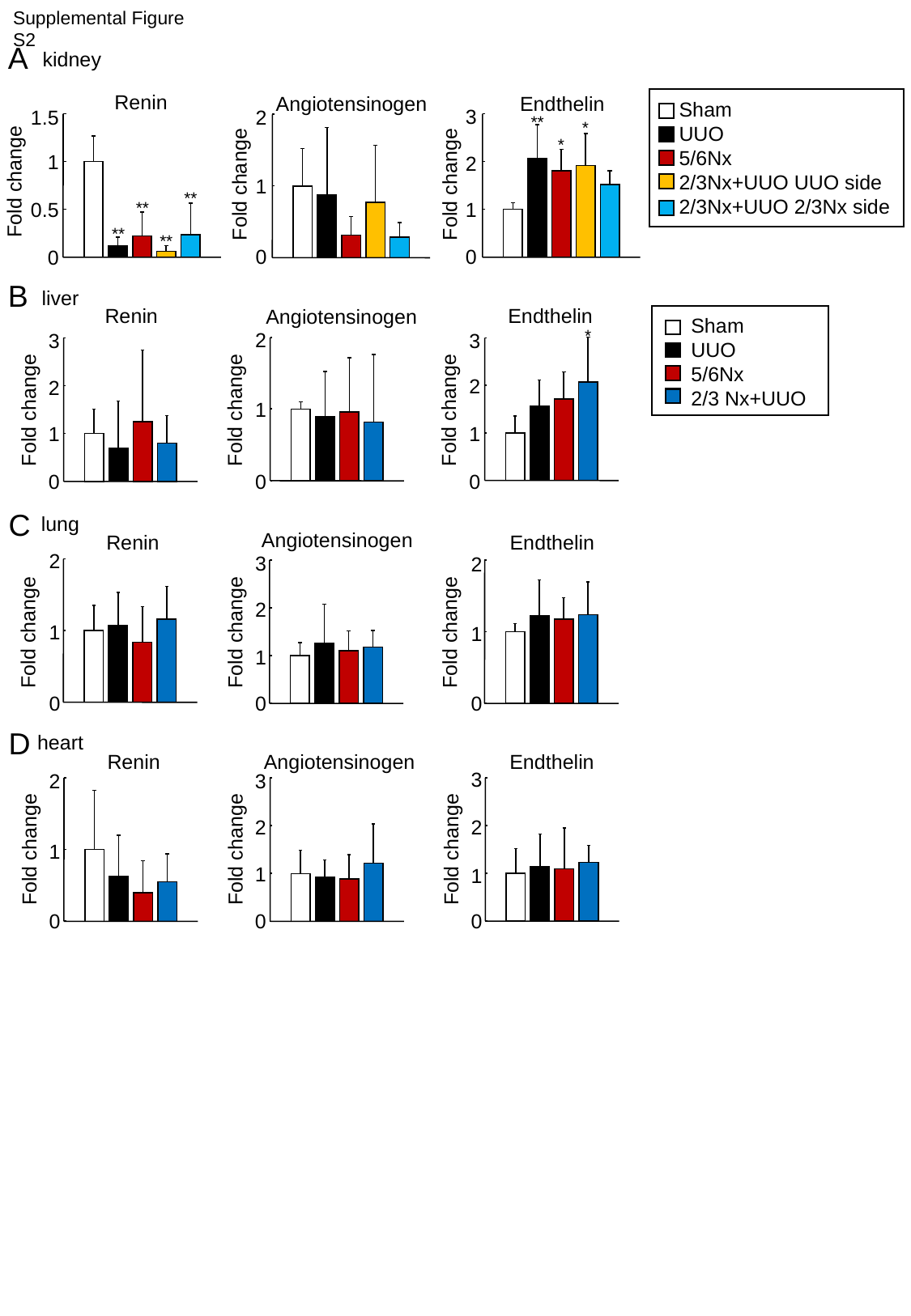

Supplemental Figure S2
A
kidney
Renin
1.5
1
0.5
0
Fold change
**
**
**
**
Endthelin
3
2
1
0
**
*
*
Fold change
Angiotensinogen
2
1
0
Sham
UUO
5/6Nx
2/3Nx+UUO UUO side
2/3Nx+UUO 2/3Nx side
Fold change
B
liver
Endthelin
Renin
Angiotensinogen
Sham
UUO
5/6Nx
2/3 Nx+UUO
*
2
3
3
2
2
1
Fold change
Fold change
Fold change
1
1
0
0
0
C
lung
Angiotensinogen
Renin
Endthelin
2
3
2
2
1
Fold change
Fold change
Fold change
1
1
0
0
0
D
heart
Renin
Angiotensinogen
Endthelin
3
2
3
2
2
Fold change
Fold change
Fold change
1
1
1
0
0
0

## Slide 3
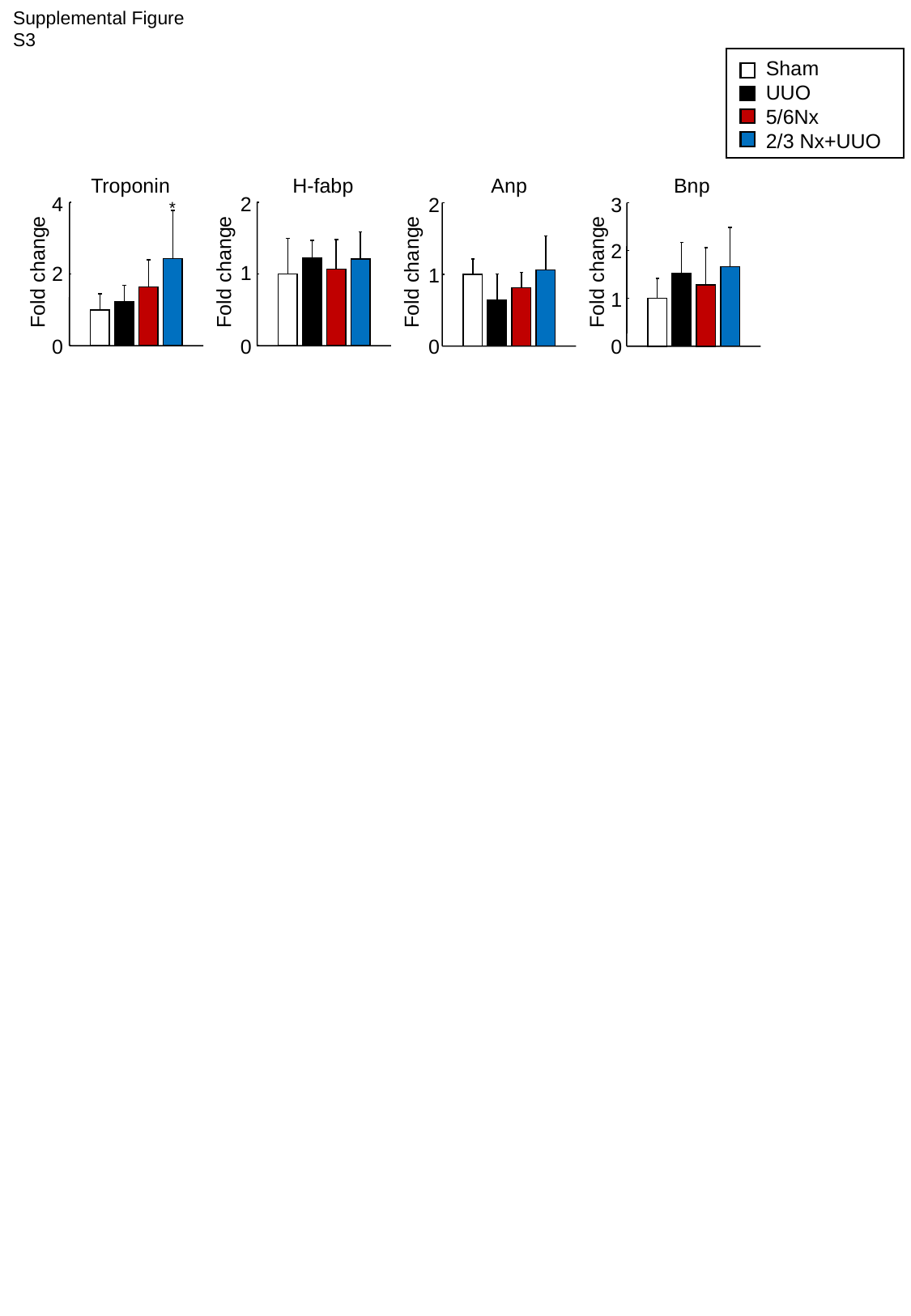

Supplemental Figure S3
Sham
UUO
5/6Nx
2/3 Nx+UUO
Troponin
4
2
0
*
Fold change
H-fabp
2
1
0
Fold change
Anp
2
1
0
Fold change
Bnp
3
2
1
0
Fold change
