# Supplementary material for: A combination of 5/6‐nephrectomy and unilateral ureteral obstruction model accelerates progression of remote organ fibrosis in chronic kidney disease
Source: FASEB Bioadv. 2023 Aug 19;5(10):377–94. doi: 10.1096/fba.2023-00045 (PMC10551277; doi:10.1096/fba.2023-00045)
Supplement: Supplementary file 3 — Data S1. [file FBA2-5-377-s002.docx]

**Supporting Information**

**Figure S1. Effect of co-stimulation of indoxyl sulfate (IS) and** transforming growth factor -β (**TGF-β) on polarization of RAW264.7 cells**

RAW264.7 cells were seeded on 96-well plates at 1.0×10^4^ cells/well and cultured overnight. After adhesion, the cells were treated with indoxyl sulphate (IS (1 mM) and TGF-β (10 ng/ml) and cultured for 72 h. Total RNA was isolated and cDNA synthesis were performed using Superprep II^🄬^ cell lysis and RT kit. mRNA expression level was quantified by real time RT-PCR using the SYBR green method. Data are expressed as the means ± SEM (n = 4). **P* < 0.05, ***P* < 0.01 compared with control.

**Figure S2. Evaluation of substances that induce renal and extra-renal damage in the three CKD models**The mRNA expression levels of renin, angiotensinogen, and endothelin-1 in the three CKD models were determined by real-time RT-PCR. (A) Kidney, (B) liver, (C) lung, and (D) heart. Data are expressed as means ± SEM (n = 5-6). *P < 0.05, **P < 0.01 compared with control.

**Figure S3. Impact of cardiac function on the three CKD models**

The effects of different CKD models on cardiac damage were evaluated 1 month after CKD induction. The mRNA expression levels of troponin, heart-type fatty acid-binding protein (h-FABP), atrial natriuretic peptide (ANP), and brain natriuretic peptide (BNP) in the heart were determined by real-time RT-PCR. Data are expressed as means ± SEM (n = 5-6). *P < 0.05, compared to the control.

**Table S1. Primers used in real-time RT-PCR**
